# Preliminary Validity and Acceptability of Motion Tape for Measuring Low Back Movement: Mixed Methods Study

**DOI:** 10.2196/57953

**Published:** 2024-08-02

**Authors:** Audrey Lee, Elijah Wyckoff, Emilia Farcas, Job Godino, Kevin Patrick, Spencer Spiegel, Rose Yu, Arun Kumar, Kenneth J Loh, Sara Gombatto

**Affiliations:** 1 Department of Bioengineering San Diego State University San Diego, CA United States; 2 Active, Responsive, Multifunctional, and Ordered-materials Research (ARMOR) Laboratory Department of Structural Engineering University of California San Diego La Jolla, CA United States; 3 Qualcomm Institute University of California San Diego La Jolla, CA United States; 4 Laura Rodriguez Research Institute Family Health Centers of San Diego San Diego, CA United States; 5 School of Public Health University of California San Diego La Jolla, CA United States; 6 Department of Mathematics and Statistics San Diego State University San Diego, CA United States; 7 Computer Science and Engineering and Halicioglu Data Science Institute University of California San Diego La Jolla, CA United States; 8 School of Physical Therapy San Diego State University San Diego, CA United States

**Keywords:** low back pain, fabric, nanocomposite, sensor acceptability, sensor validation, skin, strain, wearable

## Abstract

**Background:**

Low back pain (LBP) is a significant public health problem that can result in physical disability and financial burden for the individual and society. Physical therapy is effective for managing LBP and includes evaluation of posture and movement, interventions directed at modifying posture and movement, and prescription of exercises. However, physical therapists have limited tools for objective evaluation of low back posture and movement and monitoring of exercises, and this evaluation is limited to the time frame of a clinical encounter. There is a need for a valid tool that can be used to evaluate low back posture and movement and monitor exercises outside the clinic. To address this need, a fabric-based, wearable sensor, Motion Tape (MT), was developed and adapted for a low back use case. MT is a low-profile, disposable, self-adhesive, skin-strain sensor developed by spray coating piezoresistive graphene nanocomposites directly onto commercial kinesiology tape.

**Objective:**

The objectives of this study were to (1) validate MT for measuring low back posture and movement and (2) assess the acceptability of MT for users.

**Methods:**

A total of 10 participants without LBP were tested. A 3D optical motion capture system was used as a reference standard to measure low back kinematics. Retroreflective markers and a matrix of MTs were placed on the low back to measure kinematics (motion capture) and strain (MT) simultaneously during low back movements in the sagittal, frontal, and axial planes. Cross-correlation coefficients were calculated to evaluate the concurrent validity of MT strain in reference motion capture kinematics during each movement. The acceptability of MT was assessed using semistructured interviews conducted with each participant after laboratory testing. Interview data were analyzed using rapid qualitative analysis to identify themes and subthemes of user acceptability.

**Results:**

Visual inspection of concurrent MT strain and kinematics of the low back indicated that MT can distinguish between different movement directions. Cross-correlation coefficients between MT strain and motion capture kinematics ranged from –0.915 to 0.983, and the strength of the correlations varied across MT placements and low back movement directions. Regarding user acceptability, participants expressed enthusiasm toward MT and believed that it would be helpful for remote interventions for LBP but provided suggestions for improvement.

**Conclusions:**

MT was able to distinguish between different low back movements, and most MTs demonstrated moderate to high correlation with motion capture kinematics. This preliminary laboratory validation of MT provides a basis for future device improvements, which will also involve testing in a free-living environment. Overall, users found MT acceptable for use in physical therapy for managing LBP.

## Introduction

### Prevalence and Impact of Low Back Pain

Low back pain (LBP) is a highly prevalent and burdensome health condition, with approximately 568.4 million existing cases, 223.5 million new cases, and 63.7 million cases involving years lived with disability reported worldwide in 2019 [1]. It is anticipated that approximately 70% to 85% of adults will experience at least 1 episode of LBP during their lifetime [2,3], and once susceptible to LBP, individuals face twice the likelihood of experiencing recurring episodes [4].

The costs of diagnosing and treating LBP in the United States are substantial, collectively amounting to US $12 billion annually [5,6] and an economic impact including 149 million missed workdays per year [7]. Worldwide, the total annual costs associated with LBP are nearly US $100 billion, including lost wages and diminished productivity within businesses [8]. Given the high prevalence and burden to the individual and society, LBP is an important health condition to address clinically and in research.

### Physical Therapy for LBP

Physical therapy (PT) is effective for the conservative, nonpharmacologic, and nonsurgical management of LBP. Specifically, active interventions such as exercises prescribed by physical therapists are effective for both preventing and treating LBP [9,10]. In PT, a licensed physical therapist conducts a comprehensive initial examination to identify musculoskeletal and neuromuscular impairments associated with the LBP problem by closely observing the patient’s low back posture and movement. Subsequently, the physical therapist works with the patient to develop a plan of care for in-clinic sessions and with an assigned home exercise program based on the PT evaluation and patient goals to enhance strength, stability, and mobility [2,11,12]. These interventions collectively aim to alleviate pain and mitigate disability [13,14]. Monitoring the patient’s posture and movement, along with other patient outcomes, is an important component of the PT examination, evaluation, and intervention for LBP.

### Leveraging Technology for Posture and Movement Assessment

Traditional methods for assessing posture and movement in PT include visual assessments by clinicians or use of low-technology tools such as goniometers and inclinometers to measure gross range of motion [15]. However, advances in sensor technology allow for more detailed objective measures and enable remote monitoring [16,17]. Remote monitoring can be useful for patient assessment in free-living environments where people engage in diverse activities at home and work [18]. Quantifying the repetitive nature of specific movement patterns, whether at home or in the workplace, can help identify posture and movement factors that may be linked to the risk of developing and perpetuating LBP [19-21].

Remote monitoring of low back posture and movement can also be used to monitor patient performance of and adherence to their prescribed home exercise program. Customized by physical therapists, these home exercise programs offer practical and cost-effective management of LBP [2,7]. Adherence to and proper execution of home exercises correlate with better pain management, function, and self-perceived progress [12,22-25]. However, people with LBP have several obstacles that hinder exercise performance at home [7,11]. Impaired proprioception in patients with LBP limits their ability to sense whether they are performing home exercises accurately [15,26,27]. Moreover, the absence of clinician oversight affects patient engagement with exercises [28,29]. Previous investigators have identified that this lack of monitoring and engagement leads to diminished exercise accuracy and adherence [25].

Remote monitoring for the assessment of low back posture and movement and home exercise adherence also has the potential to enhance the emerging practice of PT via telehealth, or telerehabilitation [30,31]. Successfully implementing telerehabilitation remains challenging, primarily due to limitations in conducting movement assessments, evaluating exercise performance, and providing corrective guidance. Each of these components can be addressed using mobile sensor technologies.

### Existing Technologies for Movement Assessment

The reference standard for objective measurement of low back posture and movement is marker-based optical motion capture [32,33]. These systems offer exceptional precision and accuracy, but their use is constrained by space requirements, cost, and the expertise needed to operate them.

Several wearable and minimally invasive devices have been developed to address these limitations. In a systematic review, authors reported on various devices for measuring low back movement, which use accelerometers, electrogoniometers, gyroscopes, and strain gauges [34]. Specifically, inertial measurement units (IMUs) are commonly used portable devices for measuring lumbar spine posture and movement that use a variety of sensors, including accelerometers, gyroscopes, and magnetometers, making them well suited for capturing acceleration and orientation in real-world settings [35]. However, challenges with IMUs include their rigid structure, susceptibility to soft-tissue artifacts, misalignment, misplacement, and reduced precision during slow movements [36,37]. In addition, IMUs are not able to account for factors such as skin deformation [38] and the complex multisegmental nature of the spine [34]. Multiple IMUs are needed to evaluate spine posture and movement, which can become burdensome to the wearer [39]. Recently, flexible or fabric-based devices using piezoresistive sensors or other types of strain sensors have been used to address some of these previous limitations [37,40,41]. Table 1 shows a summary of existing sensor types for measuring low back posture and movement, characteristics that are measured, and benefits and limitations.

**Table 1:** Categories of low back sensors—characteristics, benefits, and limitations.

		Optical motion capture system	Electromyography	IMU^a^	Flexible or fabric-based sensors
**Characteristic measured**					
	Kinematics	✓^b^	✕^c^	✓	✓
	Muscle engagement	✕	✓	✕	—^d^
**Benefits and limitations**					
	Wearable	✕	—	✓	✓
	Use in free-living environment	✕	—	—	✓
	Assumption that spine segments are rigid	✓	N/A^e^	✓	✕

^a^IMU: inertial measurement unit.

^b^Yes.

^c^No.

^d^Depends on the sensor.

^e^N/A: not applicable.

### Motion Tape

Given the challenges with objective clinical assessment and the limitations of previous portable sensor systems, there is a need for an accurate, low-profile, wireless, wearable device that can be comfortably used both in the clinic and in an individual’s free-living environment to assess low back posture and movement. Motion Tape (MT) is a flexible, fabric-based sensor using commercial kinesiology tape designed to be self-adhesive and disposable [42-46]. MT has been tested on the shoulder and ankle joints in human participants [46,47] and has demonstrated the capability to measure skin strain and joint angles in the shoulder and ankle when compared to IMUs and optical motion capture systems [48]. MT has the potential to be applied to the low back and used to measure posture and movement both in the clinic and in a free-living environment.

However, the complexity in using MT for a low back use case is that the lumbar spine is multisegmental and exhibits multiplanar movements with substantial variability in skin stretch when compared to the other extremities tested previously. Therefore, these sensors must be validated for a low back use case.

### Purpose and Hypothesis

The purpose of this study was to (1) validate MT for measuring low back posture and movement and (2) assess user acceptability of MT. This is the first step in developing a use case for MT for measuring low back posture and movement. A device that is valid and acceptable in the laboratory could then be tested for use in the clinic and free-living environment for LBP diagnosis, treatment, and prevention and to further improve patient engagement and adherence to a home exercise program.

The primary hypothesis of this study was that strain-derived measures from the MT will be correlated with low back kinematics derived from a reference-standard optical motion capture system. The secondary hypothesis of this study was that users would find MT acceptable in terms of usefulness, ease of use, and wearability for the low back use case.

## Methods

### Design

**Figure 1:**
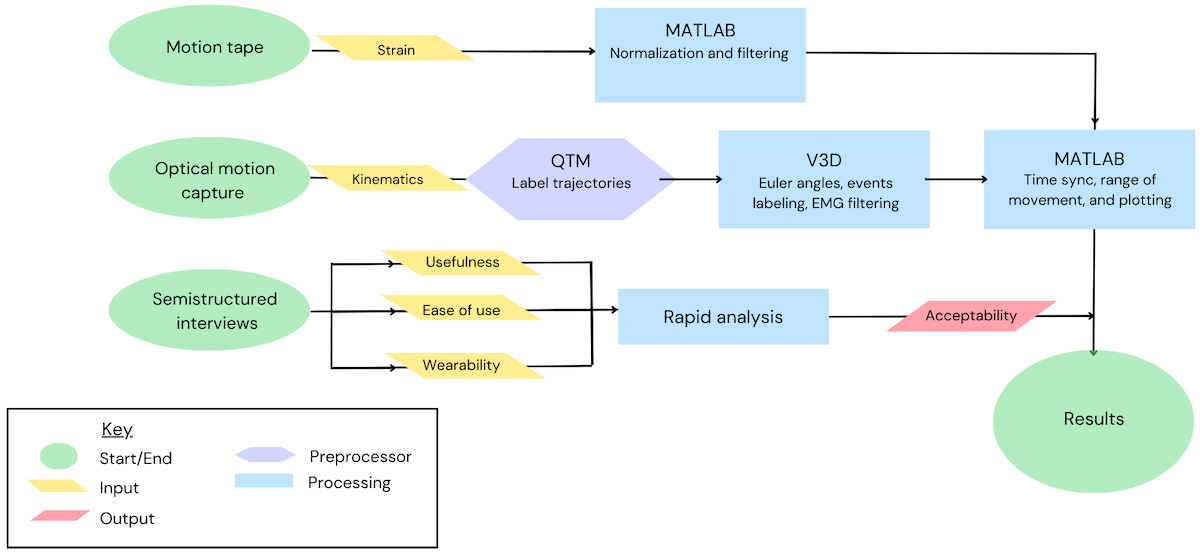
Research overview: evaluation of Motion Tape validity and acceptability. EMG: electromyography; QTM: Qualisys Track Manager; V3D: Visual3D.

This study had a cross-sectional, observational, mixed methods (quantitative and qualitative) design (Figure 1), which was used to (1) validate MT for measuring low back posture and movement and (2) evaluate user acceptability of MT using semistructured interviews. Findings from this study will provide a basis for future sensor improvements.

### Participants

A total of 10 participants were recruited from a university campus using flyers emailed to students, faculty, and staff in the kinesiology and PT programs. A sample size of 10 participants was considered adequate for a preliminary validation and acceptability study to provide a basis for improvement of the prototype device for subsequent testing in larger samples of healthy controls and people with LBP.

People were eligible to participate if they were between the ages 18 and 65 years and reported no history of LBP within the last year. People were excluded from participation if they were (1) unable to follow instructions in English; (2) unable to perform movements such as walking, sitting, and bending of the low back; and (3) unwilling to wear tight-fitting shorts and a sports bra (women) or no shirt (men). Recruitment and testing took place from January 2023 to March 2023. All data collection was conducted in the Rehabilitation Biomechanics Laboratory at San Diego State University.

### Ethical Considerations

This study was approved by the San Diego State University Institutional Review Board (HS-2022-0269), and each participant provided written informed consent before taking part. All participant data were coded, and participants were provided US $50 in compensation for their participation time.

### Equipment

MT is made by spray coating commercially available kinesiology tape with a thin film of graphene nanosheets (GNS) and ethyl cellulose (EC) in an ethyl alcohol solution 3 times [49]. To improve overall nanocomposite uniformity and electrical conductivity, a final layer of GNS and EC thin film is added through drop casting [48-50]. A flexible conductive ink is used to cover the sensor, and multistrand wires are soldered on for measurement electrodes at opposite ends of the GNS and EC sensing element [48]. MT has strain sensing due to piezoresistive properties of the integrated nanosheets in the tape [46], described in equation 1, which gives the direct relationship between measured resistance and strain. From previous research, MT has shown stable performance under cyclic strains [46,47].

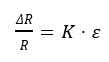



In equation 1, *R* is the resistance; *K* is the constant of proportionality, or gauge factor; and ε is the strain.

**Figure 2:**
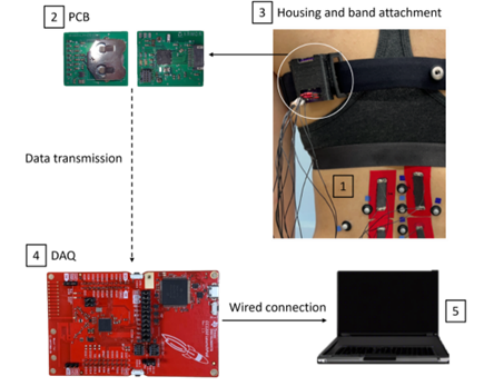
The Motion Tape (MT) system includes (1) conductive wiring that transmits the signal from the MT sensing element to a (2) custom printed circuit board (PCB) contained in (3) housing attached to the participant using an elastic band; the PCB sends a signal via a Bluetooth module transmitter to the (4) data acquisition (DAQ) board using a Bluetooth module receiver. The DAQ is then connected via micro-USB cable to (5) a laboratory computer.

The conductive wiring that attaches to the tape can directly measure distributed strains with an electrical impedance tomography measurement technique and conductivity reconstruction algorithm. The conductive wires are attached to a custom printed circuit board, which is attached to a band that can be worn on the chest or waist. The board has a Bluetooth module (Bluetooth Low Energy 4.0) transmitter, which transmits the measured signals to the MT data acquisition 2.2 board (CC1350 microcontroller; Texas Instruments), which has a Bluetooth module receiver (Bluetooth Low Energy 4.0). The MT data acquisition board was connected via micro-USB cable to the laboratory desktop computer and saved data in SmartRF Studio (version 7.1; Texas Instruments). The components of the MT system are shown in Figure 2.

An optical motion capture system (Qualisys North America, Inc) was used as the reference standard for the quantitative validation of MT. The motion capture system consists of 16 infrared cameras (sampling rate: 179 Hz) that measure the position of reflective markers on the participant’s low back and pelvis (average calibration error values: 0.57, SD 0.10 mm across all participants). Data from the MT and Qualisys software programs were collected simultaneously on the same desktop computer in the laboratory to facilitate time synchronization of measurements using alignment of start times based on time stamps in postprocessing.

### Procedure for MT Validation

#### Overview

A physical therapist investigator (SG) with >20 years of experience in motion capture of the spine located the primary anatomical landmarks of the lumbar spine (spinous processes) and pelvis (posterior superior iliac spine, anterior superior iliac spine, and iliac crests) on each participant to place the reflective markers for the optical motion capture system and the MT. The same investigator measured height, weight, and body anthropometrics for each participant. Body anthropometrics were measured in centimeters using a flexible measuring tape and included spine length (T12-S2 and L1-L5), waist circumference at the narrowest part of the waist above the iliac crests, and hip circumference at the widest part of the hips adjacent to the greater trochanter. Hip-to-waist ratio was then calculated by dividing hip circumference by waist circumference. Each participant self-reported their age and sex at birth.

#### Optical Motion Capture Marker Placement

**Figure 3:**
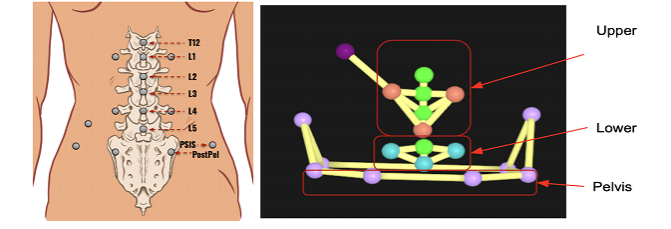
Reflective marker placement and multisegmental lumbar spine model for optical motion capture measurements. postpel: posterior pelvis; PSIS: posterior superior iliac spine.

Reflective motion capture markers were placed on the spinous processes from T12 to L5 and bilaterally to the left and right of L1 and L4 approximately 4 cm from the spinal column (Figure 3). These markers were then used to create a modified version of the multisegmental spine model that has been previously validated and used to collect lumbar spine posture and movement [51]. The upper lumbar segment was defined by the left and right markers lateral to the L1 spinous process and the single marker on the spinous process of L3. The lower lumbar segment was defined by the left and right markers lateral to the L4 spinous process and the single marker on the spinous process of L5. Markers were also placed bilaterally on the posterior superior iliac spine, anterior superior iliac spine, posterior pelvis, and iliac crests, which were used to define the pelvis segment.

#### MT Placement

**Figure 4:**
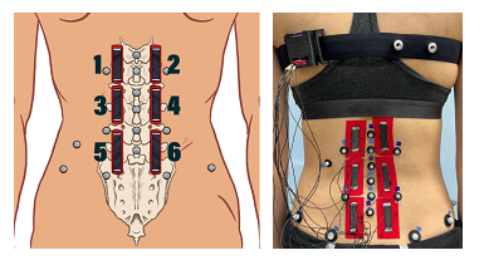
Motion Tape sensor placement.

A total of 6 MTs were placed on the low back just lateral to the spinal column in a 3 × 2 matrix pattern (Figure 4). Specifically, placement of the MTs started with the middle MTs (sensors 3 and 4) such that the bottom edges of the middle MTs were placed at a level just above the L4 spinous process and crossed the L2-to-L3 and L3-to-L4 junctions for most participants. The superior MTs (sensors 1 and 2) were placed above the middle MTs such that the superior MTs crossed the T12-to-L1 and L1-to-L2 junctions for most participants. Finally, the inferior MTs (sensors 5 and 6) were placed below the middle tapes such that the inferior MTs ideally crossed the L4-to-L5 and L5-to-S1 junctions. This placement was achieved for all but 10% (1/10) of the participants, for whom the inferior MT did not cross the L5-to-S1 junction. For this study, placement of MT was chosen to best parallel the spine model used with the motion capture system [51,52] and help distinguish low back movements in all planes of motion.

#### Measured Movements

Participants were asked to perform several simple trunk movements (forward flexion, extension, right and left lateral flexion, and right and left seated rotation) while data were simultaneously being captured by the motion capture system and the MT (Figure 4). The complete list of tested movements is shown in Table 2.

**Table 2:** Trunk movements, positions, repetitions, and range of movement.

Movement	Position	Repetitions
Lateral bending	Standing	3 repetitions to end range on each side (left and right)
Rotation	Seated	3 repetitions to end range on each side (left and right)
Extension	Standing	3 repetitions to approximately 50% of end range^a^
Forward flexion	Standing	2 repetitions to approximately 50% of end range^a^ and 1 repetition to 100% of end range

^a^50% range was used to avoid maximum capacity of sensors before the end of the session.

### Data Processing for MT Validation

#### Overview

Kinematic data from the optical motion capture system were processed in Qualisys Track Manager (Qualisys North America, Inc) to label marker trajectories and interpolate missing marker data. Kinematic data were then imported into Visual3D (C-Motion, Inc), where a previously developed multi-segmental spine model (Figure 3) was applied and lumbar spine kinematic angles were computed for each movement trial [51]. Lumbar spine kinematic angles were calculated using Euler angles (XYZ sequence) among the upper lumbar, lower lumbar, and pelvis segments (Textbox 1). Processed kinematic angles were then imported into MATLAB (release 2021b; MathWorks) for analysis with MT strain data.

**Kinematic measurements from the optical motion capture system.**

**Lumbar spine angle and relative segments**

- Upper lumbar angle: upper lumbar segment (L1-L3) relative to lower lumbar segment (L4-L5)
- Lower lumbar angle: lower lumbar segment (L4-L5) relative to pelvis segment

Raw resistance data from the 6 MTs were imported into MATLAB and converted from hexadecimal characters to decimal values. The change in resistance was divided by the baseline resistance individually for each sensor and each movement trial to derive strain (equation 1). Resistance values were then read and stored in an array where time vectors were generated linearly from start to end using time stamps. Once all MT resistance files were imported, stored, and converted to readable time series, they were filtered using a Hampel filter to remove outliers. The filter is based on the median and median absolute deviation of the data set. For some MT placements and some movements (eg, lower MTs during forward flexion), MT stretch exceeded resistance thresholds for the sensing element, resulting in data with excessive levels of noise. These data streams were identified and removed using a threshold criterion of resistance of >10 SDs from the mean resistance across participants for the given movement.

**Figure 5:**
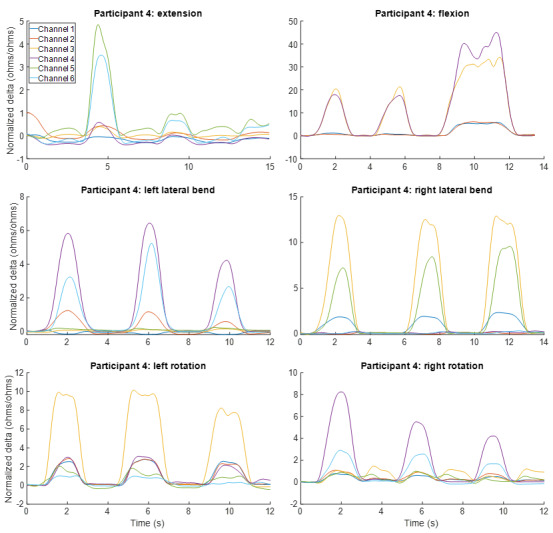
Motion Tape strains for all movements for a representative participant.

To illustrate the ability of MT to capture data across all test movements, strain measured using the 6 MTs for 1 representative participant is illustrated in Figure 5. Data for MTs 5 and 6 are omitted for forward flexion because the stretch during this movement exceeded the MT strain threshold for this participant.

Motion capture kinematic data and MT strain data were aligned in MATLAB using the computer time stamp for the start of each trial from the motion capture system. Excess data at the start and end of the trial for strain were then trimmed to ensure identical start and end times for kinematics and strain. MT strain data and motion capture kinematic data were normalized separately for each trial to allow for the use of MATLAB’s cross-correlation function. The strain data were normalized from –1 to 1 such that –1 corresponded to peak sensor compression and 1 corresponded to peak sensor tension. The strain-derived data were then shifted such that each movement started at zero strain. Kinematic data were also normalized from –1 to 1 such that –1 and 1 corresponded to peak movement in each direction. For analysis purposes, the normalized kinematic data from forward flexion, left lateral bending, and right-seated rotation were multiplied by –1 such that all kinematic measurements were positive for the primary movement direction (eg, upper lumbar flexion is a positive angle for the forward flexion movement).

#### Analysis for MT Validation

In previous studies, MT has been validated to measure strain using ground truth input from a TestResources 100R load frame, where resistance was recorded using a Keysight 34401A digital multimeter [48]. This study used an accepted reference standard (optical motion capture) for validating kinematic measurements using MT. Cross-correlation was used to test concurrent validity of MT strain in reference to motion capture kinematics [53]. Cross-correlation is a measure of the association between 2 data series as a function of the time displacement (phase shifts) of one relative to the other. Strain data from the 6 MTs were compared to motion capture kinematics for adjacent low back segments, as outlined in Textbox 2. Cross-correlation coefficients at zero phase shift were derived to ensure that both the magnitude and timing of MT strain were considered for evaluation of concurrent validity. Coefficients were calculated separately for each participant, movement trial, and MT. Positive cross-correlation values reflect MT tension with changes in kinematic angle, and negative values reflect MT compression with changes in kinematic angle. Median values and range of cross-correlation coefficients at zero phase shift were calculated across all participants.

**Lumbar spine kinematics used as reference for validating Motion Tape.**

**Motion Tapes (Figure 4) and lumbar spine angle (Figure 3)**

- 1 and 2: upper lumbar angle
- 3 and 4: upper lumbar angle
- 5 and 6: lower lumbar angle

### Procedure for User Acceptability

#### Semistructured Interviews

To assess user acceptability of MT, semistructured interviews were conducted with all participants (N=10) after laboratory testing. A semistructured interview guide (Multimedia Appendix 1) was developed by investigators based on the technology acceptance model (TAM) [54-56]. The guide included open-ended questions designed to evaluate user perceptions of MT in 3 key domains of the TAM: usefulness, ease of use, and wearability. Perceived *usefulness* was defined as the extent to which participants believed that using MT could improve treatment of LBP [54-56]. Specific interview questions related to (1) potential advantages of using MT in PT treatment and recovery, (2) potential impact of MT use on adherence to home exercise programs, and (3) physical attributes of MT that could positively or negatively affect its usefulness. Perceived *ease* *of use* was defined as the extent to which participants believed that using MT would be effortless for evaluation and treatment of LBP [54-56]. This domain was evaluated using questions related to participants’ perceptions regarding (1) potential ease of learning to use MT, (2) level of instruction required for effective use of MT, and (3) ease of using MT unsupervised in a home setting. *Wearability* was defined as the extent to which participants believed that MT sensors provided a comfortable and secure fit when applied to their back [57]. To assess wearability, interview questions explored participants’ views on various aspects of MT, including its adhesion, fit, feel, and comfort level with the application and prescription of MT by a medical professional to monitor posture and movements at home. Finally, additional interview questions were included to gather participant suggestions for future improvements of MT. Interviews were recorded using digital voice recorders and transcribed for subsequent analysis.

#### Analysis for User Acceptability

Rapid qualitative analysis (RQA) was conducted to assess the interview responses effectively and efficiently to identify major themes [58]. Codes and themes for the RQA were deductively developed based on the TAM framework and the study objective. The codes and themes for the RQA allowed for quick sorting of interview dialogue. To ensure rigor and consistency, a constant comparative approach was used at each stage. First, the 4 data analysts independently completed a summary report for each interview with quotes and relevant topics under identified themes. Once the individual coding and summary reports for all interviews were completed, the investigators consolidated them into a combined RQA summary report for each interview, unifying themes and reconciling discrepancies by consensus through discussion. Summary reports for each participant were then transferred into a matrix where each row was a participant quote and each column was a domain. From this matrix, investigators identified underlying themes and subthemes across the 10 interviews.

## Results

### Demographics

A total of 10 people participated in the study (n=5, 50% male and n=5, 50% female; mean age 22.4, SD 2.1 y). Participant ages and anthropometric measurements are presented in Table 3.

**Table 3:** Participant age and anthropometric measurements.

Demographics	Male participants (n=5)	Female participants (n=5)
Age (y), mean (SD)	23.6 (2.9)	21.2 (1.8)
Height (in), mean (SD)	70.9 (3.7)	64.2 (4.0)
Weight (pounds), mean (SD)	178.8 (41.6)	125.1 (11.4)
Hip-to-waist ratio (cm), mean (SD)	1.2 (0.04)	1.3 (0.3)
Spine length (T12-S2; cm), mean (SD)	16.8 (1.9)	16.4 (1.3)
Spine length (L1-L5; cm), mean (SD)	10.7 (1.2)	9.7 (0.6)

### MT Validation

**Figure 6:**
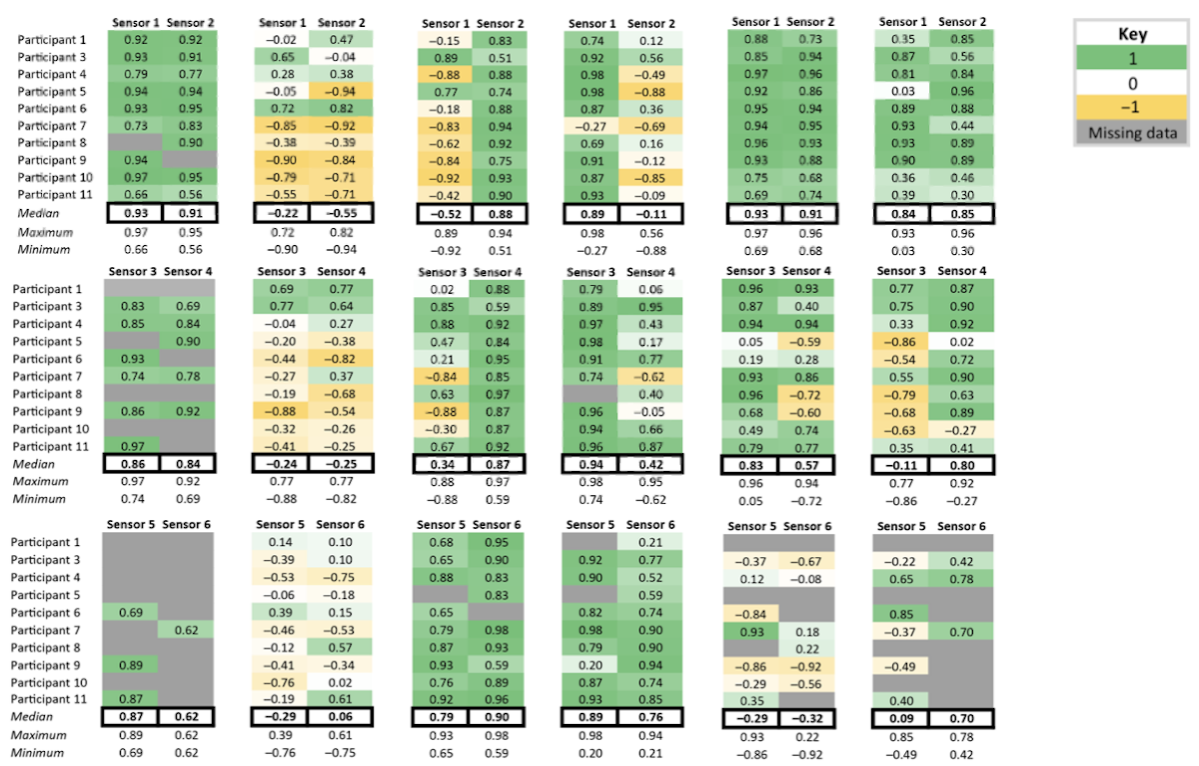
Cross-correlation values at zero phase shift for low back Motion Tape strain versus motion capture kinematics. The level of correlation is depicted using a color scale, with green denoting a positive correlation with a maximum of +1 (Motion Tape tension), yellow denoting a negative correlation with a maximum of –1 (Motion Tape compression), and no color for cross-correlation values near 0. Shades of each color reflect magnitudes of correlation, with lower correlations in lighter colors and higher correlations in darker colors. Trials with missing data are colored in gray.

Values for cross-correlation coefficients at zero phase shift between MT and motion capture low back kinematic measurements across the 6 movements for all 10 participants are presented in Figure 6. Across movement trials, 13.9% (50/360) of MTs had missing data because resistance exceeded the threshold of 10 SDs. There are two potential explanations for why sensors exceeded the resistance threshold: (1) the level of strain for the low back region exceeded the capacity of the sensor and (2) sensor resistance increased across trials due to sensor fatigue, resulting in high resistance values even at lower strains. The former was most common during flexion movements, and the latter occurred more often in trials near the end of the testing protocol, such as rotation movements.

### Forward Flexion Movement

For *forward* *flexion* movements, cross-correlations were mostly positive (green) and moderate to high (median 0.62-0.93), indicating that MT sensors were in tension and closely paralleled motion capture kinematic measures during forward flexion (Figure 6). However, there was a high rate of sensor failure for flexion movements (45%; 27/60), particularly for lower lumbar MTs.

**Figure 7:**
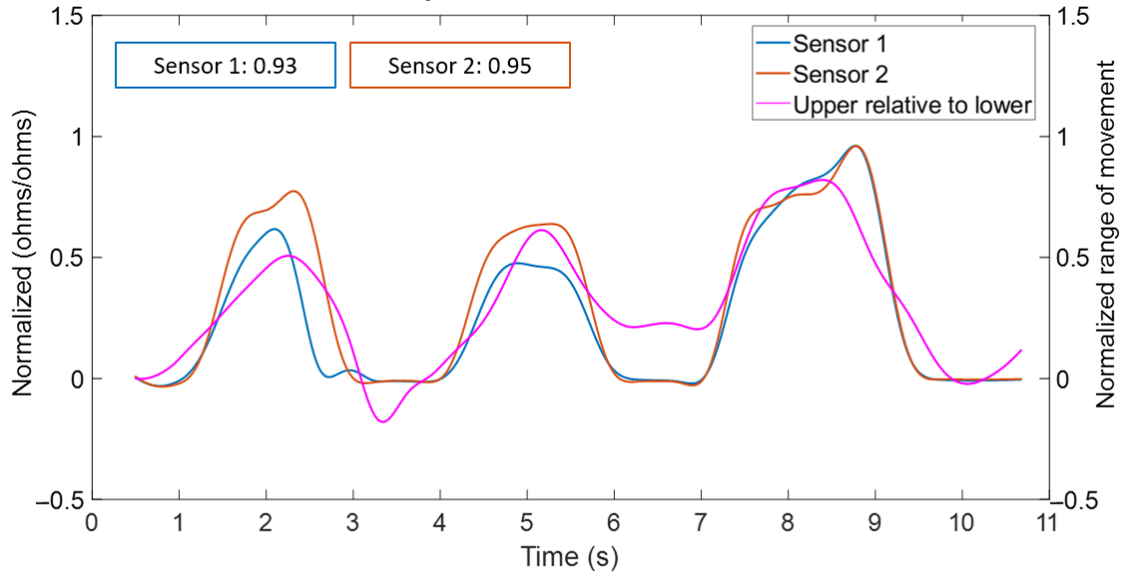
Case example of high positive cross-correlation between Motion Tape strain for Sensor 1 and 2 and motion capture upper lumbar angle during forward flexion.

To illustrate the association between MT strain and motion capture kinematics during forward flexion, data for sensor 1, sensor 2, and the upper lumbar angle are shown in Figure 7 for a single participant. In this example, upper lumbar MT strains are highly correlated with the upper lumbar angle (*R*=0.94 for sensor 1 and *R*=0.95 for sensor 2). These positive correlations reflect MT tension, which was consistent for all forward flexion movements.

### Extension Movement

For *extension* movements, many cross-correlation coefficients were negative, indicating MT compression. However, there were also several positive cross-correlations that had varying magnitudes. This resulted in many cross-correlations that were high in magnitude for individual MTs and participants but median values that were low (median –0.55 to 0.06), indicating that strain measures closely paralleled kinematic measures but were sometimes in tension and sometimes in compression.

**Figure 8:**
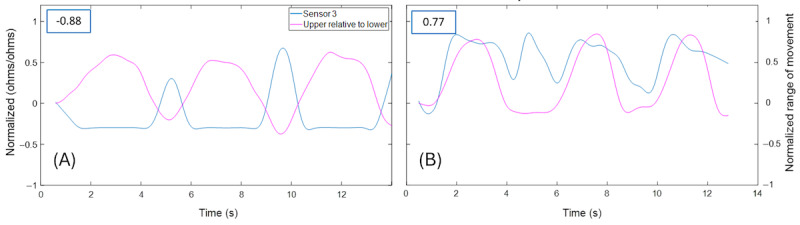
Case examples of different cross-correlation values between sensor 3 strain and motion capture upper lumbar angle for 2 different participants during extension.

To illustrate varied patterns of MT strain when compared to motion capture kinematics during extension, Figure 8 shows sensor 3 strain data and upper lumbar angle measures for 2 different participants who performed extension. For the first participant (left), MT measured compression (negative deflection) during the extension movement, resulting in a high negative cross-correlation value (*R*=–0.88). For this participant, sensor 3 also appeared to show a limit in the ability to measure maximal compression values, as evidenced by a flattening of the strain curve at peak extension. The second participant (right) showed an unexpected pattern during extension, in which MT measured tension (positive deflection) during the extension movement, resulting in a high positive cross-correlation value (*R*=0.77). For both participants, an increase in MT strain was also evident when the participant was returning to an upright position from the extension movement, which did not appear to align with the decrease in the kinematic measures.

### Lateral Bending Movements

For *right and left lateral bending movements*, cross-correlation coefficients were positive and high (median 0.87-0.94) on the side opposite the direction of the lateral bend, indicating that MT was typically in tension and closely paralleled kinematic measures during the lateral bend movements. Cross-correlation coefficients for MT sensors on the side ipsilateral to the lateral bend movement were more variable (median –0.52 to 0.79). This illustrates that some ipsilateral sensors (upper) were in compression during the trunk lateral bending movement but correlations were low to moderate, whereas other sensors (lower) were in tension and showed high positive correlations. The middle sensors on the side ipsilateral to the lateral bending movement showed participant-to-participant variability in both direction and magnitude of cross-correlations.

**Figure 9:**
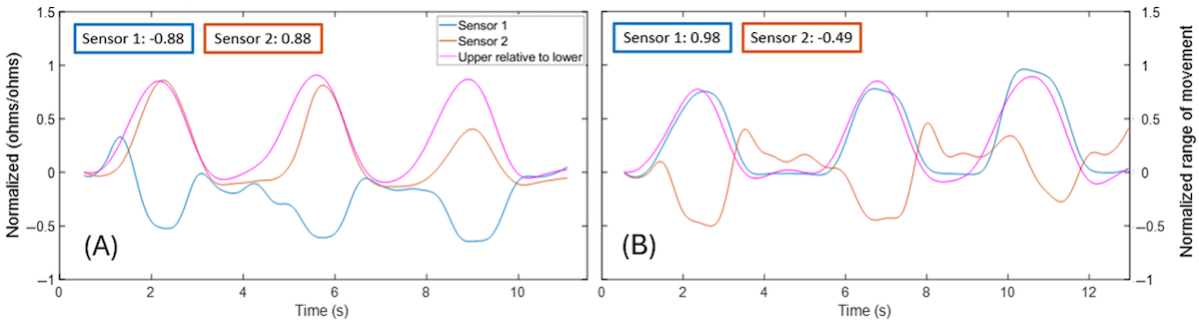
Case example of upper sensor positively correlated (tension) with upper lumbar angle on the contralateral side and negatively correlated (compression) on the ipsilateral side during left (A) and right (B) lateral bending.

**Figure 10:**
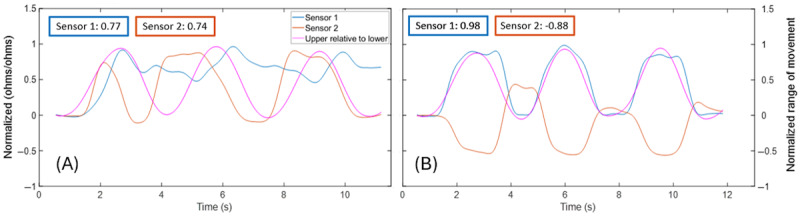
Case examples of positive correlation (tension) between bilateral upper sensors and upper lumbar angle during left (A) lateral bending, but not with right (B) lateral bending.

Figure 9 illustrates a case example of the expected MT strains for right and left lateral bending, in which the ipsilateral MT strain is negatively correlated (compression) and the contralateral MT strain is positively correlated (tension) with the motion capture upper lumbar angle. In contrast, Figure 10 illustrates a case example of a positive correlation between MT strain and motion capture kinematics on both sides of the low back during left lateral bending, suggesting that both MTs were in tension during this movement. However, during right lateral bending for the same participant, the expected MT tension on the contralateral side and compression on the ipsilateral side were observed.

### Rotation Movements

For *right and left rotation movements*, cross-correlation coefficients were positive and high (median 0.84-0.93) for both upper MTs for both movement directions, indicating tension and strong association with motion capture kinematics. Cross-correlation coefficients for middle and lower MTs were more variable (median –0.11 to 0.83). Most middle sensors were in tension on the side *ipsilateral* to the rotation movement, and MT strain was highly correlated with motion capture kinematics (median 0.80-0.83 for sensors 3 and 4). However, on the side contralateral to the rotation movement, the middle sensors showed wide participant-to-participant variability in both direction and magnitude of cross-correlations. Cross-correlations between lower sensors and motion capture kinematics varied widely on the sides both ipsilateral and contralateral to the rotation movement (median –0.32 to 0.70).

**Figure 11:**
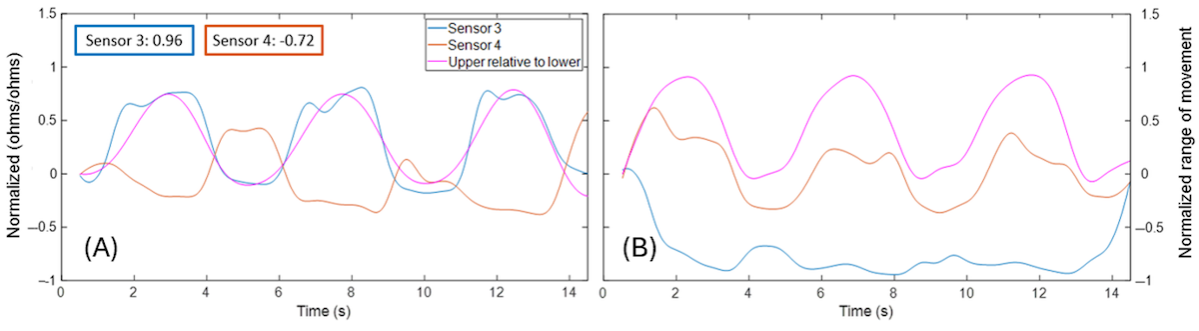
Case example of ipsilateral positive correlation (tension) and contralateral negative correlation (compression) between middle sensors and upper lumbar angle during seated left (A) rotation and right (B) rotation.

**Figure 12:**
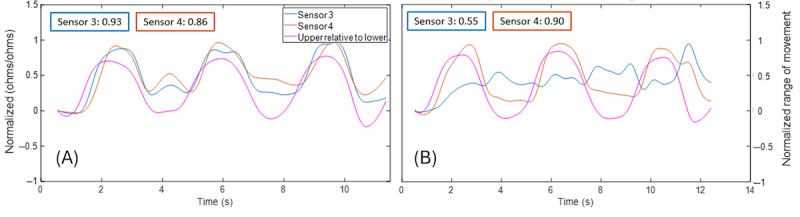
Case example of bilateral positive correlation (tension) between middle sensors and upper lumbar angle during seated left (A) rotation and right (B) rotation.

Figure 11 illustrates a case example for data from middle MTs (sensors 3 and 4) for right and left rotation for a participant, in which the ipsilateral MT exhibited a positive correlation (tension) and the contralateral MT exhibited a negative correlation (compression) with the upper lumbar angle for both rotation directions. In contrast, a second case example (Figure 12) illustrates middle MTs that were both positively correlated with the upper lumbar angle during both left and right rotation, suggesting that both sensors were in tension during these movements.

### User Acceptability

#### Overview

Qualitative results from participant interviews on user acceptability of MT were organized based on the domains of the TAM, including perceived wearability, perceived usefulness, and perceived ease of use (Table 4) [54-56]. A total of 13 subthemes were also identified and designated as having a “positive,” “negative,” or “neutral” valence. Positive subthemes were those that the participants perceived as a positive attribute of MT, negative subthemes were those perceived by participants as negative, and neutral subthemes were those perceived as neither positive nor negative.

**Table 4:** Themes (n=3), subthemes (n=13), and valences of user acceptability of Motion Tape (MT).

Theme, valence, and subthemes				Respondents (n=10), n (%)
**Theme 1: perceived wearability**				
	**Positive valence**			
		MT has secure adhesive properties.	10 (100)	
		MT removal process is not painful.	10 (100)	
		MT is a good fit on low back anatomy.	9 (90)	
		MT causes minimal discomfort and is not very noticeable during low-intensity movements.	8 (80)	
	**Negative valence**			
		Concerns with MT’s wiring and attachment band	7 (70)	
		Awareness of MT may limit ROM^a^ and exercises for some people.	4 (40)	
**Theme 2: perceived usefulness**				
	**Positive valence**			
		MT may offer positive benefits for use in physical therapy.	10 (100)	
		Potential benefits for MT use in telehealth	10 (100)	
		MT could increase patient adherence to and motivation to perform home exercise programs.	5 (50)	
		MT offers benefits for personalized medicine.	5 (50)	
	**Negative valence**			
		Overall concerns about MT usability and durability	6 (60)	
**Theme 3: perceived ease of use**				
	**Neutral valence**			
		MT is easy to use but may be difficult for a patient to apply themselves.	6 (60)	
		Mixed perceptions on whether one could use the MT on their own at home	6 (60)	

^a^ROM: range of motion.

#### Domain 1: Wearability

Regarding perceived *wearability*, most participants were familiar with commercially available kinesiology tape. Thus, their thoughts on perceived wearability reflected both their experience with kinesiology tape and their experience wearing MT during laboratory testing. Generally, participants felt comfortable wearing MT and would feel comfortable if a medical professional prescribed MT for them to wear. All participants felt that MT was secure on their back during validation testing. One participant stated that the tape was “pretty secure and stretched with your body.”

Participants predicted that the tape would remain adhered on their back for approximately 2 to 3 days depending on various factors such as the level of activity, temperature, and moisture. One participant gave an example of how the adhesive properties would change:

If you worked out or did something really physical it could get less sticky over time, but I think that the tape is pretty stable otherwise.

Regarding the *fit* of MT, most participants felt that it was a good size and did not hinder their movements. They noted that it adhered closely to their skin, was not bulky, and could be stored easily. However, one taller participant mentioned the following:

Since I’m a taller individual, some strips weren’t long enough for my back.

Regarding the *feel* of the MT on their skin, most participants were aware of its presence but generally found it comfortable and unobtrusive; one participant stated the following:

It didn’t feel like it was in the way of anything, and it didn’t feel like it was there.

While they could feel a slight pull on their skin during movements with larger ranges, this was not perceived as a significant problem. One participant said the following:

When I was bending down [flexion], I could feel it more. But otherwise, it wasn’t that bad. I kind of got used to it.

Regarding *awareness* of MT while exercising, participants had varied responses. Most perceived that the tape would not impede their exercise performance, but some had concerns. They noted being aware of and concerned about damaging or dislodging the sensor wires during exercises, especially with intense workouts. They generally preferred a wireless design and found that the MT wires were “messy,” “hard to handle,” and “somewhat restrictive.” In addition, participants anticipated that they would feel the sensors on their bodies, especially when their clothing rubbed against them. One participant thought the following:

The Motion Tape sensor’s adhesion is pretty strong, but if it started to peel off, then I might be more aware of not letting it come off.

A few participants also expressed concerns about the attachment band for the MT system, particularly around the chest. They believed that this feature might be uncomfortable for larger or female individuals. Regarding removal of the MT, participants reported minimal pain and discomfort; several participants likened the sensation to removing a Band-Aid and did not find it very painful.

#### Domain 2: Perceived Usefulness

Regarding a *PT use case,* participants felt that the MT offered several useful benefits. For example, several participants agreed that this would be helpful for identifying the cause of pain and give medical providers that information. A few participants mentioned that the MT could be used by the physical therapist:

To monitor stress that’s being put on a specific part of the back and spine and figure out a way to adjust or to alleviate some of that pain and tension.

To give much more insight into what I am actually feeling.

To track the patterns of your movement and recruitment of your muscles, and check if there is any irregularity.

In addition, participants expressed that the MT could offer continuous monitoring for them even when the physical therapist is not present, allowing for better patient management. One participant suggested that it could detect if “you’re moving a certain way that could be further injuring you.”

Most participants felt that the use of MT to monitor and record their PT exercises would serve as a good reminder or external cue to increase their adherence to their prescribed home exercise program. Participants noted that having their exercises recorded and monitored would provide them with more motivation to do their exercises and perform them more regularly and correctly. For example, one participant stated that they would “probably do the PT exercises more regularly, especially since it’s being recorded. Can’t really lie about that.”

Furthermore, participants expressed that MT offers advantages for personalized medicine and precise data on back pain. Participants felt that the MT would be helpful for pain management, injury rehabilitation, and providing a better understanding of movement. One participant stated that MT “offers an opportunity to measure movement of the human body in a new way [for treatment and recovery].”

Furthermore, participants expressed that it would also be particularly useful for older people who are not able to make appointments with their provider:

For the older patients who aren’t able to make their doctor’s appointments, if they had [motion] tape applied to them and then they were sent home, I think it would be pretty easy for them...

Thus, participants generally felt that the MT was beneficial and advantageous for monitoring movements in a free-living environment to assist with PT management.

Regarding a *telehealth use case*, participants felt that there was some potential usefulness for MT. Some participants expressed that they perceived the use of MT for telehealth more convenient and easier than attending in-person PT appointments. Participants predicted that there would be an increase in remote visits because they felt that the MT would allow them to be more independent and do PT on their own time without having to schedule in-person appointments and leave their homes. One participant explained the following:

It would save people a trip outside, or if they were busy, they could just do it whenever they could, instead of having to schedule an appointment. I think it could definitely benefit people.

Participants also expressed that they could envision MT increasing their compliance and adherence to therapy, leading to better outcomes. They felt that the MT would allow the physical therapist to see what is going on and whether patients are performing their exercises correctly, which would lead to increased engagement of the patient in their own treatment and incentivizing adherence to the home exercise program. One participant explained the following:

Patients would feel like they’re more involved in the treatment, rather than just the PT evaluating them over a call and then telling them exercises to do.

However, a few participants did not feel that MT would increase remote PT sessions. For example, one participant expressed concerns regarding the use of the MT with older individuals as the older generations may find it challenging to use the technology and some prefer in-person visits with a physical therapist. In addition, another participant expressed that, while the device may be helpful on days when in-person visits are not possible, some individuals still prefer to use the equipment available in the clinic. Therefore, while there are potential benefits, the use of the device and technology for telehealth may not be suitable for everyone and should be carefully considered on a case-by-case basis.

#### Domain 3: Perceived Ease of Use

Regarding the *application process* for MT, participants felt that MT would be easy to use but difficult to apply to one’s own back. Specifically, participants expressed that older or less flexible individuals would struggle in applying it to their back. One participant stated the following:

Grandma would struggle, but someone mobile enough wouldn’t struggle after getting thorough instructions and doing it a couple of times.

Perceptions of the application process also affected how the participants felt about whether the average person would be able to use the MT on their own at home. Some participants indicated that they would prefer that a physical therapist apply it to their back, whereas others felt that they would be able to apply MT themselves if shown how to apply it appropriately.

Regarding the *use* of MT, participants expressed that it would be generally easy to use but they would also need detailed instructions on how to use it properly. Participants suggested a variety of instructional methods, including written instructions, pictures, videos, in-person visits, and demonstration by a physical therapist; visuals and demonstrations were emphasized as most important. Participants also noted that they needed information on the calibration process, how to turn the sensors on, how to charge the sensor, how to reapply the tape if it falls off, how to care for the tape or reattach wires if they fall off, and whether the tape is safe to wear in water. There were some concerns expressed about ease of use. Specifically, some participants mentioned that lack of access to technological support could make it difficult for some individuals to use the MT without assistance.

## Discussion

### MT Validation

MT demonstrated the ability to measure low back movement in multiple directions and in a manner comparable to that of a reference-standard motion capture system. Cross-correlations between MT strain and motion capture kinematic measures were moderate to high for most movement directions and appeared to better reflect kinematics for movement directions in which MT was in tension (Figure 6). Patterns of MT strain appeared different for different low back movements (eg, flexion, extension, lateral bending, and rotation), suggesting that MT can distinguish between different movement directions (Figure 5).

However, for several movements and sensors, there was variability in magnitude and direction of association between MT strain and motion capture kinematics. Figures 8-12 show case examples that demonstrate variability in direction (positive vs negative) of cross-correlations during extension, lateral bending, and rotation movements. Variability in direction of association, which reflects MT tension (positive) versus compression (negative), may be the result of differing movement strategies performed by each participant. As an example, Figure 10 illustrates a positive correlation between MT strain on *both* sides of the low back and the lumbar angle during *left* lateral bending (tension bilaterally) but a positive correlation only on the *contralateral* side (tension) and a negative correlation on the *ipsilateral* side (compression) during *right* lateral bending. These data may suggest that lateral bending movements are performed, in some cases, by lengthening the spine (Figure 10; bilateral tension with left lateral bending) and, in other cases, by compressing or pivoting at spinal segments (Figure 10; tension and compression with right lateral bending). Low back kinematics during lateral bending were not different between sides for this case example, suggesting that MT was able to capture a level of data that is different from motion capture, which could be useful for identifying new impairments in people with LBP.

A limiting factor of the existing lower back sensing technologies summarized in Table 1 is that some can only capture movement in a single plane [59]. Other existing devices that can capture multiple planes of movement often rely on more rigid sensors [38,60]. Ensuring that the device seamlessly integrates with the wearer’s natural movements and environment is a common challenge faced for wearable sensor technology [61]. MT provides a cost-effective solution for capturing kinematics in multiple planes and that has the potential for longer-term use in a free-living environment [60]. MT’s capability to stretch and conform with the skin sets it apart from other fabric-based and flexible sensors, providing more comprehensive measurement of lumbar posture and movement [37,51,62,63]. Therefore, MT holds the potential to become a valuable tool for the assessment, treatment, and monitoring of LBP.

### User Acceptability

Several key themes emerged related to the wearability, usefulness, and ease of use of MT. Concerning wearability, participants observed that MT securely adhered to their backs during the validation testing, and they anticipated that it would stay in place for approximately 2 to 3 days, with some variation due to external factors. This aligns with the typical time frame of use for commercially available kinesiology tape, estimated to last for 2 to 3 days [57,64]. The flush-with-skin fit and feel were perceived as not likely to disrupt daily activities, but participants expressed concerns about the wired design, the chest band attachment, and potential friction between clothing and the sensors. The current MT system design, with wires and a chest band attachment, may not be optimal [61]. Previous research has highlighted the widespread adoption and use of *wireless* technologies in various fields, particularly in the domain of health care wearable devices [58]. Therefore, a future iteration of MT that minimizes the wires and chest band attachments would be ideal to improve user perceptions of wearability.

Regarding usefulness, participants believed that MT had the potential to enhance personalized PT treatment and, importantly, serve as a helpful reminder to engage in and adhere to prescribed exercises. Devices that allow for remote monitoring of patients have the potential to broaden the scope of assessments, enhance treatment outcomes, and enable physical therapists to make informed decisions for future patients [61]. Nevertheless, certain design limitations might hinder the usefulness of this device by older, less flexible, or larger individuals. Our findings align with earlier research studies emphasizing the need for wearable sensors to be not only useful and convenient but also inclusive and accessible to a diverse population [65]. Therefore, future iterations of MT should address inclusivity and accessibility concerns to enhance user perceptions of usefulness.

The *ease of use* of a wearable device is closely intertwined with its usability and the user’s confidence in its correct operation [62]. Regarding ease of use, participants acknowledged that applying MT might be challenging without assistance, but they anticipated that it would be straightforward if accompanied by detailed instructions. Providing comprehensive information about the device fosters confidence and competence in its correct use, leading to reduced errors and improved user acceptability [63,66]. Failing to provide adequate use instructions could result in the incorrect use of MT, potentially adversely affecting patient outcomes and decreasing user acceptability.

Overall, participants expressed enthusiasm and curiosity regarding the innovative nature of MT and believed that it could offer more personalized and insightful treatment for LBP, particularly due to its potential for remote monitoring. However, they also highlighted certain aspects that would require attention in future iterations to enhance user acceptability.

### Limitations and Opportunities for Future Research

The participants in this study were primarily university students in exercise and nutritional science programs who were young and fit, may have more knowledge of low back anatomy and PT, and may be more inclined to accept new technologies than people from other demographics. Collectively, this negatively impacts the generalizability of the findings to other clinical populations. Older adults or individuals with obesity may display different skin strains due to differing characteristics of skin and subcutaneous fat, which could impact the validity of MT measurements. In addition, patients with LBP may display limited movement or different movement characteristics, which were not tested in this study. Furthermore, MT may be less acceptable to older patients, who may have a preference against use of technology as part of PT treatment. However, starting with validity testing in healthy young participants allowed for testing of the full range of movement for MT measures, which may not be possible in other populations. The standardized verification, analytical validation, and clinical validation for biometric monitoring technologies recommends conducting analytical validation in a healthy population first, followed by validation in a clinical population [67]. Future research is needed to test MT acceptability and validity for measuring low back movement in a more diverse patient population, including people of different ages, with a variety of body types, and with LBP. Comparing results between people with and without LBP will also help differentiate movement patterns between the 2 groups.

In addition to assessing patient user acceptability, it is important to evaluate provider acceptability for use of new technologies in clinical practice. While this study did not assess provider acceptability of MT, we conducted a preliminary study to evaluate physical therapist acceptability of MT, and these findings are reported elsewhere [68]. As a first step in validating MT for a low back use case, this study was limited to a laboratory environment. Future studies including sensor improvements and development of a mobile app will allow the MT system to be used and tested for acceptability and validity in a free-living environment.

Because of their standard size, the location of MTs relative to spine anatomy may be slightly different for each person. As previously mentioned, the inferior MTs (5 and 6) were placed below the middle tapes such that the inferior MTs ideally crossed the L4-to-L5 and L5-to-S1 junctions. However, for 1 taller participant, the inferior row was not long enough to span the L5-to-S1 junction. Therefore, an additional limitation may be variable strain readings due to variability in sensor placement relative to participant anatomy. Our study team is currently investigating the impact of variability in placement on skin-strain measurements.

MT performed well when measuring mid ranges of movement that resulted in tension on the sensor but was limited in its ability to measure maximal tension and compression. Daily tasks are rarely performed at end ranges of movement; thus, the ability to distinguish movement in mid ranges may be the most critical for ecological monitoring. However, future sensor iterations will focus on increasing limits for measuring maximal tension and compression. Second, there were some instances of increases in MT strain that do not appear to correspond with motion capture kinematics (eg, Figure 8; return from extension). It is possible that this increase in strain could reflect increased muscle engagement associated with the phase of movement. Because MT measures skin strain, it may have the potential to detect changes in skin strain as a result of muscle engagement. The instances of MT strain that did not correspond with motion capture kinematics may indicate that the MT detected another physiological phenomenon, such as muscle engagement. This has been demonstrated empirically in other areas of the body, including the biceps and gastrocnemius muscles [48]. Research is currently underway to investigate the extent to which MT has the capability to capture muscle engagement in the low back.

It is also possible that increases in MT strain that do not correspond with motion capture kinematics may be due to a sensor rebound effect. Preliminary laboratory tests conducted by investigators confirmed a rebound effect when a compressed MT returns to a neutral position (Wyckoff E, unpublished data, February 2024). Due to the piezoresistive property of MT, the resistance may momentarily increase due to a delayed mechanical relaxation of the GNS and EC ink matrix, causing a temporary increase in the distance between conductive pathways. This rebound effect has also been observed in piezoresistive carbon [69]. The rebound effect could explain some of the lower correlations observed for movements that result in compression of MT. Following compression movements, there were positive strain values that did not correspond to the kinematics but, rather, may reflect this rebound effect. Additional research is needed to investigate the extent and true nature of the rebound effect and determine how this effect can be accounted for in measures of low back strain.

### Conclusions

In this study, MT demonstrated moderate to high association with most low back motion capture kinematic measurements and can distinguish among multiple directions of movement. The median cross-correlation values were highest for lateral bending (0.87-0.94) and rotation (0.84-0.93) but varied more during forward flexion (0.62-0.93). For movements with expected positive correlations (MT tension), the highest correlations were observed in the upper MTs, 1 and 2 (0.84 and 0.80, respectively). However, several measurement limitations exist for the current version of MT, including limited ability to measure compression as demonstrated by poor to moderate median cross-correlation values for extension movements (–0.55 to 0.06). The MT also demonstrated limited capacity for measuring maximal tension associated with end ranges of certain movements (eg, flexion). User acceptability assessment indicates primarily positive feedback in the domains of perceived wearability and usefulness but more equivocal feedback related to ease of use in its current form. Future sensor developments and testing will be focused on addressing these issues.

## Appendix

Multimedia Appendix 1 A semistructured interview guide based on the technology acceptance model.

## Abbreviations

EC: ethyl cellulose
GNS: graphene nanosheets
IMU: inertial measurement unit
LBP: low back pain
MT: Motion Tape
PT: physical therapy
RQA: rapid qualitative analysis
TAM: technology acceptance model

## Data Availability

The data sets generated during and analyzed during this study are available from the corresponding author on reasonable request.
