# Supplementary material for: Preliminary Validity and Acceptability of Motion Tape for Measuring Low Back Movement: Mixed Methods Study
Source: JMIR Rehabil Assist Technol. 2024 Aug 2;11:e57953. doi: 10.2196/57953 (PMC11329853; doi:10.2196/57953)
Supplement: Multimedia Appendix 1 [file rehab_v11i1e57953_app1.pdf]

<u>INTERVIEW GUIDE</u>

Kinesiology Tape Sensor Feedback Survey

Introductions

Interviewer:
Good morning/afternoon. My name is ______________________________. The purpose of this interview is to obtain your feedback about our Kinesiology-Tape ("K-tape") Sensors.

We have asked you to take part in this interview because you have participated in the laboratory testing of these K-Tape Sensors and could help us get a better understanding about how people would react to them. We want to learn from you. Please feel free to give us your honest feedback about the K-Tape Sensors even if you do not like them. Your ideas and opinions are important as we continue to improve these sensors for use in the community.

Our interview today will last approximately 1 hour. It is informal, and there are no right or wrong answers. Your participation is voluntary. If you feel like you need a break or want to stop, please let me know. The information that you tell me today will be kept private. A final report will be prepared using the information that you give me, but your name and other identifying information will not be used. We hope that you feel comfortable to speak freely.

For this interview, I have several questions to ask you. We know that your time is important, so we want to be sure to keep you only for the time that we promised. To make that happen, I may ask you to finish your thought or sentence and then move on to another topic or question.

I will ask if I may tape record our conversation so that I can pay more attention to you and what you have to say instead of trying to write everything down. We will transcribe our interview from this tape. Is it okay for me to begin recording now?

[If permission given, begin recording.]

Date: ______________________________ Participant #: ______

Say into audio recorder: This is (*Name of Researcher*). Today is (*date*), and I am interviewing

participant number (*Participant Number*).

Thank you so much for taking part in the interview. Are you ready to begin?

1. First impression question:

   Recall the beginning of this testing session when K-Tape was shown to you and placed on your back.

   a. *What was your first impression of the K-Tape Sensors?*

2. Wearability: an added variable to the TAM [KEY: W, Factors of Analysis: (1) adhesion, (2) fit, (3) feel, (4) application/prescribing]

   I would now like you to answer the following questions regarding the wearability of these K-Tape sensors. Wearability is the degree to which you believe the sensors fit well and are comfortable to wear on your back.

   a. *How secure was the K-Tape adhesive on your back? [W_1]*
      i. *PROBE: How secure do you think the K-Tape adhesive will be if worn for an entire day/several days?*
   b. *How well did the K-Tape sensors fit on you back? [W_2]*
      i. *PROBE: Was the tape too big/small for your back?*
   c. *How much did you feel the K-Tape on your back during testing? [W_3]*
      i. *PROBE: How well do you think the K-Tape would feel under your normal clothes while you are about your normal routine?*
      ii. *PROBE: Do you think your level of awareness affected your range of movement? Why or why not? [W_3]*
      iii. *PROBE: Do you think your level of awareness would affect your performance of exercises? Why or why not?*
   d. *How did the K-Tape sensors feel when being removed? [W_3]*
   e. *How comfortable would you feel if you were prescribed K-Tape by a medical professional to monitor your movements at home? [W_4]*

3. Perceived usefulness: [KEY: TAM_U, Factors of Analysis: (1) work more quickly, (3) increase productivity, (4) effectiveness, (5) makes job easier, (6) useful]

   I would now like you to answer the following questions regarding the usefulness of these K-Tape sensors. Usefulness is the degree to which you believe that using these sensors would enhance the treatment of low back pain.

   a. *How do you think the sensors could be used to help with treatment/recovery of low back pain? [TAM_U1,4,3]*
   b. *To what degree would having the sensors monitor your movements affect your performance of at-home exercises? [TAM_U5]*
   c. *To what degree to you find the physical features of the K-Tape useful to your treatment of lower back pain? [TAM_U6]*
      i. *PROBE: Can you list some features that* ***make*** *the K-Tape sensors useful/effective in your treatment of lower back pain? [TAM_U4,6]*
      ii. *PROBE: Can you list some features that* ***do not*** *make the K-Tape sensors useful/effective in your treatment of lower back pain? [TAM_U4,6]*
   d. *What features, if any, would make the K-Tape* ***more*** *useful to you? [TAM_U6]*
      i. *PROBE: Why or why not?*

4. Perceived ease of use: [TAM_EU, Factors of Analysis: (1) easy to learn, (2) clear and understandable~~,~~ (3) easy to use]

   I would now like you to answer the following questions regarding the ease of use of these K-Tape sensors. Ease of use is the degree to which you believe using these sensors would be free of effort when using for treatment of low back pain.

   a. *How easy do you think it would be to learn how to use the K-Tape sensors? [TAM_EU1]*
   b. *What level of instruction do you think an individual would need to use the K-Tape?* [TAM_EU3]
   c. *Do you think you would be able to use the K-Tape sensors at home on your own? [TAM_EU1,6]*
      i. *PROBE: Why or why not?*

5. Concluding questions:
   a. *What kind of benefits, if any, do you think K-Tape can offer?*
      i. *PROBE: Why?*
   b. *What kind of limitations, if any, do you think K-Tape presents?*
      i. *PROBE: Do you think wearing the K-Tape would pose any concerns?*
         1. *Probe: Why or why not?*
      ii. *PROBE: What, if anything, would you like to see improved about the K-tape sensor?*
         1. *PROBE: Why or Why not?*
   c. *If this sensor was offered along with remote physical therapy (by phone or video), do you predict patients would utilize PT remote services more often?*
      i. *PROBE: Why or why not?*

<u>More Response Probing</u>

a. *How have you reached this answer?*
b. *How did you arrive to that answer?*
c. *What made you say that?*
d. *Is there anything else you would like to add?*
e. Restate their answer. For example, *"So you believe you would be able to complete your normal daily routine, despite wearing the K-Tape Sensors?"*
f. *Can you take me through the steps of how you came to that answer?*

6. When done with the interview, say to the participant*:*
"Thank you so much for taking part in the interview. I am going to stop recording now".
